# The Influence of Odors upon the Voice

**Published:** 1896-04

**Authors:** 


### The Influence of Odors upon the Voice.

"It is well known to singers," says *Popular Science News*, "that perfumes influence the voice. The violet is regarded by artists as the flower which especially causes hoarseness. The rose, on the contrary, is regarded as inoffensive. M. Joal, who has studied the subject, says he does not believe that the emanations of the violet prevent free vibrations of the vocal cords, and thinks that if this flower has any injurious effect upon the voice, the rose and other flowers must have the same action. There is, in fact, nothing fixed or regular in the influence exerted by the perfume of flowers. It is a matter of individual susceptibility. Some are affected by the lilac; others by the mimosa. Others, again, are in no manner affected by flowers, musk, amber, civet, or the various toilet preparations, but experience obstruction of the nose, hoarseness and oppression, from the odors of oils, grasses, burnt horn, and the emanations from tanneries and breweries. It is very difficult, adds M. Joal, to furnish an explanation of these peculiarities, and we must content ourselves by regarding them as examples of olfactory idiosyncrasy. It cannot be denied that odors may occasion various accidents and vocal troubles, especially in persons of nervous temperament and excessive sensibility."—*Literary Digest.*

